# Aberrant Expression of High Mobility Group Box Protein 1 in the Idiopathic Inflammatory Myopathies

**DOI:** 10.3389/fcell.2020.00226

**Published:** 2020-04-17

**Authors:** Jessica Day, Sophia Otto, Kathy Cash, Preethi Eldi, Pravin Hissaria, Susanna Proudman, Vidya Limaye, John D. Hayball

**Affiliations:** ^1^Experimental Therapeutics Laboratory, University of South Australia Cancer Research Institute, Adelaide, SA, Australia; ^2^School of Pharmacy and Medical Sciences, University of South Australia, Adelaide, SA, Australia; ^3^Royal Adelaide Hospital, Adelaide, SA, Australia; ^4^SA Pathology, Adelaide, SA, Australia; ^5^Discipline of Medicine, University of Adelaide, Adelaide, SA, Australia; ^6^Robinson Research Institute and Adelaide Medical School, University of Adelaide, Adelaide, SA, Australia

**Keywords:** myositis, idiopathic inflammatory myopathy, HMGB1, immune-mediated necrotising myopathy, immune-mediated necrotizing myopathy, inclusion body myositis, necrotising autoimmune myopathy, necrotizing autoimmune myopathy

## Abstract

**Introduction:**

High Mobility Group Box Protein 1 (HMGB1) is a DNA-binding protein that exerts inflammatory or pro-repair effects upon translocation from the nucleus. We postulate aberrant HMGB1 expression in immune-mediated necrotising myopathy (IMNM).

**Methods:**

Herein, we compare HMGB1 expression (serological and sarcoplasmic) in patients with IMNM with that of other myositis subtypes using immunohistochemistry and ELISA.

**Results:**

IMNM (*n* = 62) and inclusion body myositis (IBM, *n* = 14) patients had increased sarcoplasmic HMGB1 compared with other myositis patients (*n* = 46). Sarcoplasmic HMGB1 expression correlated with muscle weakness and histological myonecrosis, inflammation, regeneration and autophagy. Serum HMGB1 levels were elevated in patients with IMNM, dermatomyositis and polymositis, and those myositis patients with extramuscular inflammatory features.

**Discussion:**

Aberrant HMGB1 expression occurs in myositis patients and correlates with weakness. A unique expression profile of elevated sarcoplasmic and serum HMGB1 was detected in IMNM.

## Introduction

The idiopathic inflammatory myopathies (IIMs) are a group of systemic autoimmune diseases characterized primarily by muscle inflammation, but also potentially accompanied by a range of extra-muscular manifestations. In adults, the term encompasses dermatomyositis (DM), polymyositis (PM), inclusion body myositis (IBM) and immune-mediated necrotising myopathy (IMNM; also called necrotising autoimmune myopathy, NAM). The etiology of these conditions remains obscure and the pathogenic mechanisms likely differ between the subtypes, given their distinct histopathological and immunological features. Immune-mediated necrotising myopathy has been only relatively recently described and the molecular mechanisms underlying the immune attack on muscle are poorly understood (Allenbach and Benveniste, 2013; Allenbach et al., 2018). It is becoming increasingly apparent that a dysregulated innate immune system contributes to the initiation and perpetuation of the IIMs, with roles for type I interferon (IFN), toll-like receptors (TLRs), various cytokines and the alarmin, High Mobility Group Box Protein 1 (HMGB1), now well-established (Day et al., 2017).

HMGB1 is a ubiquitous non-histone nuclear DNA-binding protein that can, under certain physiological and pathological conditions, undergo extra-nuclear translocation where it may act as a signal of tissue damage and a pro-inflammatory mediator (Lotze and Tracey, 2005). In response to injurious stimuli, inflammatory cells actively secrete HMGB1 in a controlled manner, which requires post-translational modification of the protein (Gardella et al., 2002; Lu et al., 2014). HMGB1 is also rapidly passively released from necrotic cells, following nuclear membrane breakdown (Scaffidi et al., 2002). This protein is implicated in a broad range of conditions, including sepsis, malignancy and autoimmune diseases (Harris and Raucci, 2006; Diener et al., 2013; Magna and Pisetsky, 2014). With regards to IIM, extra-nuclear HMGB1 expression has been demonstrated in the muscle of mice with experimental autoimmune myositis (Wang and Qin, 2016) and in muscle from IBM (Muth et al., 2015), PM, and DM (Ulfgren et al., 2004; Grundtman et al., 2010) patients. HMGB1 positive fibers in PM and DM muscle are non-necrotic, suggesting active release of HMGB1 from the muscle cell nucleus (Ulfgren et al., 2004). Of note, sarcoplasmic HMGB1 expression in IIM patients has not been confirmed in all reports (Cseri et al., 2015). Patients with new-onset DM and PM have elevated serum HMGB1, and these levels correlate with survival and the presence of interstitial lung disease (ILD) (Shu et al., 2016). In addition to descriptive research, a pathogenic role for HMGB1 in muscle is suggested by experimental studies. For instance, myocytes or myofibres exposed to recombinant HMGB1 demonstrate intracellular protein aggregation, increased cell death (Muth et al., 2015), aberrant MHC-I expression (Grundtman et al., 2010; Muth et al., 2015) and impaired calcium release during repeated tetanic stimulation, suggesting enhanced muscle fatigue (Grundtman et al., 2010; Zong et al., 2013).

However, HMGB1 is a multifaceted protein that exerts different effects depending on its redox state, the presence of post-translational modifications, the complexes it forms with other stimulatory or inhibitory proteins and the cellular receptor through which it ultimately signals. HMGB1 has been shown to induce muscle regeneration in mouse models of ischaemic myopathy (De Mori et al., 2007) and a non-oxidisable mutant form of exogenous HMGB1 promotes muscle and liver regeneration in mice via interaction with the CXCR4 receptor (Tirone et al., 2018). Cytosolic HMGB1 is a crucial regulator of autophagic responses to cellular stress, where autophagy is a beneficial physiological process enabling cellular proteins to be degraded and recycled (Tang et al., 2010). Within IIM muscle, HMGB1 co-localizes marker of autophagy (Cappelletti et al., 2014). As such, while HMGB1 appears to play a role in IIM pathophysiology and may have direct negative effects on muscle function, it may paradoxically aid in muscle restoration. The role of HMGB1 in IIM appears complex and clearly warrants further investigation. Moreover, research evaluating expression of this protein in IIM has focused on DM, PM and IBM and these discoveries may not be readily extrapolated to the condition of IMNM.

Herein we compare expression of HMGB1 in IMNM patients with that of other IIM patients. We correlate these findings with clinical, histopathological and serological parameters. To our knowledge, this is the largest cohort study evaluating HMGB1 in IIM and the first to describe sarcoplasmic and serum levels of HMGB1 in IMNM.

## Materials and Methods

### Subjects

Muscle tissue, serum and clinical data were obtained from the South Australian Myositis Database (SAMD), a registry of patients with PM, DM, IBM, non-specific IIM (NSIIM) and ‘necrotising myopathy.’ Recruitment to the SAMD is based on histological criteria which have previously been described for PM, DM, and IBM (Limaye et al., 2013). All cases of PM, DM and IBM adhered to published classification criteria (Hoogendijk et al., 2004; Rose and Group, 2013). Cases are recorded as ‘necrotising myopathy’ if there is myofibre necrosis and an absence of histological features consistent with other neuromuscular conditions. This was considered to be immune-mediated if the treating clinician documented a diagnosis of IMNM or NAM. Cases of NSIIM have muscle inflammation with insufficient biopsy criteria to allow subclassification (e.g., scattered perimysial or endomysial inflammation that does not surround or invade myofibres). Muscle from 62 patients diagnosed with IMNM between 2001 and 2016, was included. Forty-five patients with a diagnosis of DM, PM, or IBM were included for comparison in addition to 15 patients with NSIIM and 17 controls. Control muscle constituted biopsies obtained from subjects with diffuse myalgia, weakness or unexplained creatine phosphokinase (CK) elevations, but which lacked any myopathic features. Control serum samples were collected from healthy volunteers. Some cases were excluded from this study due to lack of clinical information, alternative diagnosis or technical difficulties (Supplementary Figure 1).

Clinical information was prospectively recorded. This included data regarding the presence of weakness, dysphagia, extramuscular features and exposure to medications such as statins. As part of standard clinical assessment, most patients were tested for the following myositis-specific and myositis-associated antibodies (MSAs, MAAs; Euroline Myositis Profile 3): anti-signal recognition peptide (SRP), anti-histidyl-tRNA synthetase (Jo1), anti-threonyl-tRNA synthetase (PL7), anti-alanyl-tRNA synthetase (PL12), anti-glycyl-tRNA synthetase (EJ), anti-isoleucyl-tRNA synthetase (OJ), anti-Mi2, anti-Ro52, anti-Ku, anti-PMSCl75 and anti-U1RNP. A subset were analyzed for anti-3-hydroxy-3-methylglutaryl-CoA reductase (HMGCR) antibodies (ELISA, PathWest). Disease activity and damage were assessed in a number of patients using the following: (1) Manual Muscle Testing 8 (MMT8), (2) Patient and physician global assessments by visual analog scale (VAS; PtG and DrG respectively), (3) Health Assessment Questionnaire (HAQ) Disability Index, (4) the muscle activity component of the Myositis Disease Activity Assessment Tool by VAS (MDAAT-muscle), (5) CK and (6) clinician assessment of muscle damage by VAS (Myositis Damage Index; MDI). The date of symptom onset and the degree of corticosteroid exposure at biopsy were collected retrospectively. This study was approved by the Central Adelaide Local Health Network Ethics Committee and the University of South Australia Human Research Ethics Committee.

### Muscle Biopsy

Muscle samples were obtained via open surgical biopsy or needle biopsy. Specimens were placed into transverse orientation, mounted on cork, frozen in isopentane cooled using liquid nitrogen (Cash and Blumbergs, 1994) and stored in liquid nitrogen until sectioning. Consecutive 9 μm-thick cryostat muscle sections were placed on coated slides. H&E staining was performed on the first and last sections of each series. Sections intended for MHC I, MHCn, CD68, CD45 and LC3 staining were air dried for 30 min then stored at −80°C until use (1 – 68 days). Sections intended for HMGB1 staining were air dried for 30 min, fixed for 10 min in 10% neutral buffered formalin, air dried for 30 min then stored at −80°C until use (1 – 33 days). On the day of staining, antigen retrieval was achieved on slides intended for HMGB1 staining by immersion in sodium citrate buffer (pH 6.0) followed by heat induced epitope retrieval utilizing microwave treatment.

### Immunohistochemistry

Immunohistochemistry was performed using an autostainer. Slides were washed in buffer (TA-999-TT, Thermo Scientific), blocked for 5 min with 2% H_2_O_2_ in methanol, washed again with buffer then incubated for 5 min with a commercial blocking agent (TA-060-PBQ, Thermo Scientific). After a further wash with buffer, slides were incubated with primary antibody at room temperature for 30 min. Primary antibodies used were anti-HMGB1 at 1/300 (ab18256, Abcam), anti-MHC I at 1/2000 (M0736, Dako), anti-CD45 at 1/400 (M0701, Dako), anti-CD68 at 1/8000 (M0814, Dako), anti-CD8 at 1/75 (M7103, Dako), anti-LC3 at 1/50 (AM1800a, Abgent), anti-neonatal myosin heavy chain (MHCn) at 1/100 (NCL-MHCn). Antibodies were diluted in commercial antibody diluent (TA-125-ADQ, Thermo Scientific). Slides were then washed, incubated in commercial primary antibody enhancer (TL-060-PB, Thermo Scientific) for 10 min, washed and incubated with a commercial detection system comprising a universal secondary antibody formulation with anti-Mouse IgG and anti-Rabbit IgG specificity, conjugated to horse-radish-peroxidase polymer (TL-060-PH, Thermo Scientific) for 15 min. After washing in buffer, slides were incubated with peroxidase-compatible liquid substrate chromagen system (Dako K3468) for 10 min, followed by haematoxylin counterstaining.

Positive controls were performed for each antibody in every staining procedure. A negative control was performed by omitting primary antibody in every staining procedure, both for an unfixed slide and a formalin-fixed microwave-retrieved slide. For ten cases, a formalin-fixed, antigen-retrieved section underwent staining with a rabbit IgG isotype control (Invitrogen, 31235), at the same concentration as the HMGB1 antibody. Lymphoid tissue was used to confirm HMGB1 staining in non-muscle tissue.

### Modified Gomori Trichrome Staining

Consecutive 9 μm-thick cryostat sections of unfixed fresh frozen IBM muscle, normal muscle and a positive “ragged red” control were stained with Harris’ haematoxylin for 30 min, washed then stained with modified Gomori trichrome stain (laboratory-prepared, pH 3.4) for 15 min followed by differentiation with 0.2% aqueous acetic acid.

### Quantification of Immunohistochemical Staining

Slides were graded using traditional microscopy for HMGB1, MHC I, LC3 and MHCn staining in a semi-quantitative manner by a muscle pathologist (SO) (Supplementary Table 1). The H&E stained sections were graded for degree of necrosis by the same pathologist, where necrosis was defined as muscle cells exhibiting a combination of the following features: swelling, hyalinization, hypereosinophilia, pallor, myophagocytosis. Slides were graded by twice on separate occasions; median grades are reported. A second trained investigator (JD) validated the grading scale by manually counting positive and negative myofibres in 10 randomly selected high power fields (HPFs, magnification × 400) and calculating the percentage of HMGB1+ fibers. Manual cell counts of CD45+ leucocytes and CD68+ macrophages were also performed. Evaluation of each histological parameter was completed for the entire cohort before proceeding to the next parameter. At the time of histological evaluation, investigators were blinded to the clinical details and the grades assigned to other immunoproteins.

### Serum Collection and Analysis

A commercial ELISA kit was used to measure serum concentrations of HMGB1 (Cloud-Clone Corporation, Texas, United States). Each sample was diluted 1/100 in phosphate buffered saline and tested in duplicate. One sample was re-tested on each ELISA plate.

### Statistics

Statistical analysis was performed using STATA version 14.0. Values were expressed as the median and the interquartile range (IQR). Two group comparisons were performed using the Mann–Whitney *U* test. When three or more groups were compared, a Kruskal–Wallis *H* test was conducted to identify whether a statistically significant difference existed, followed by a *post hoc* Dunn’s test. A Bonferroni correction was applied for multiple comparisons. Fisher’s exact test was used to analyze categorical data. Spearman correlations were performed to analyze associations between radiological grades and continuous or ordinal parameters. Number of cases analyzed are indicated if a full data set was unavailable. *P*-values < 0.05 were considered significant.

## Results

### Immunohistochemistry Grading Reliability

Intra-rater reliability was high (κ > 0.70) for all histological parameters. The average percentage of HMGB1+ myofibres correlated strongly with grades assigned by the muscle pathologist (*r*_s_ 0.83, *p* < 0.01).

### Subject Characteristics

#### Subjects Undergoing Immunohistochemical Analyses

Clinical characteristics of subjects are presented in Table 1. Fifty-eight patients had both serum and muscle tissue available for analysis. DM patients were more likely to have received corticosteroids at the time of biopsy (*p* = 0.002); this may reflect more frequent occurrence of extramuscular IIM features in this subgroup. Serum from IIM patients was collected within 139 days (56–695 days) of the muscle biopsy and most (71%; 40/56) were on immunotherapy at the time of venepuncture. Compared with other IIM subsets, patients with PM or DM were more likely to exhibit extramuscular manifestations such as rash (*p* < 0.001), Raynaud’s phenomenon (RP, *p* < 0.01), inflammatory joint disease (*p* = 0.03), ILD (*p* = 0.02) and to be MAA-positive (*p* = 0.01). Fifty percent (56/113) of the pooled serum and IHC cohort were MSA+ and/or MAA+, and a variety of antibodies were represented (Supplementary Table 2). Presence of anti-Ro52 was most common (*n* = 23), followed by anti-HMGCR (*n* = 10), anti-PL7 (*n* = 7), anti-Mi-2 (*n* = 5), anti-Jo1 (Jo1+, *n* = 5), anti-SRP (SRP+, *n* = 5), anti U1RNP (*n* = 3), anti-PL-12 (*n* = 3), anti-PMSCL75 (*n* = 3), anti-PMSCl100 (*n* = 2), anti-Ku (*n* = 1), and anti-OJ (*n* = 1). Two IMNM patients were anti-Mi2+ but had myonecrosis on biopsy and lacked clinical features or histopathology consistent with DM. One patient was anti-HMGCR+, but had minor necrosis and inflammatory histopathology consistent with PM.

**TABLE 1:** Subject characteristics.

	IMNM	DM	PM	IBM	NSIIM	Controls
**Subjects Undergoing Histological Analysis**						
*N*	62	18	13	14	15	17
Age (years)^a^	64 (57 – 69)	55 (47 – 70)	60 (45 – 66)	68 (64 – 79)	59 (47 – 69)	45 (26 – 51)
Female	32 (51%)	12 (67%)	7 (54%)	7 (50%)	10 (67%)	10 (59%)
Symptom Duration (Days) ^a^	47	104	136	1275		UA
	(22 – 176)	(88 – 274)	(122 – 244)	(1096 – 2718)	92 (75 – 130)	
	*n* = 50	*n* = 8	*n* = 9	*n* = 9	*n* = 5	
MSA positivity	18/44 (41%)	5/16 (31%)	3/13 (23%)	2/12 (17%)	2/12 (17%)	UA
MAA positivity	4/44 (9%)	8/17 (47%)	6/13 (46%)	2/12 (17%)	3/13 (23%)	UA
EM feature^b^	10/58 (17%)	17 (94%)	7 (54%)	5 (36%)	9/14 (64%)	NA
PNL dose (mg)^a^	0 (0 – 0)	20 (0 – 55)	0 (0 – 7)	0 (0 – 0)	0 (0 – 0)	0 (0 – 0)
	*n* = 48	*n* = 12	*n* = 9	*n* = 13	*n* = 14	*n* = 15
Cumulative PNL dose (mg)^a^	0	840	0	0	105	UA
	(0 – 0)	(150 – 2625)	(0 – 370)	(0 – 0)	(0 – 350)	
	*n* = 35	*n* = 12	*n* = 8	*n* = 13	*n* = 9	
Peak CK (IU/L)	4086	546	2237	470	400	164
	(1467 – 10799)	(248 – 1942)	(897 – 3193)	(217 – 709)	(73 – 839)	(92 – 673)
MMT8^c^	76 (64 – 80)	70 (62 – 80)	74 (64 – 80)	64 (54 – 74)	80 (78 – 80)	NA
	*n* = 23	*n* = 7	*n* = 7	*n* = 8	*n* = 5	
**Subjects Undergoing Serological Analysis**						
*N*	34	14	10	13	5	50
Age (years)^d^	65 (57 – 70)	67 (48 – 75)	60 (52 – 72)	67 (67 – 78)	69 (69 – 70)	45 (29 –56)
Female	15 (44%)	8 (57%)	8 (80%)	7 (54%)	4 (80%)	20 (40%)
Days since biopsy^d^	185	93	141	119	190	UA
	(51 – 1687)	(54 – 677)	(85 – 805)	(59 – 181)	(0 – 334)	
MSA positivity	7/24 (29%)	4/12 (33%)	2/10 (20%)	1/10 (10%)	1/4 (25%)	UA
MAA positivity	3/24 (13%)	7/13 (54%)	4/10 (40%)	3/10 (30%)	1 (20%)	UA
Cumulative PNL dose (mg)^d^	3250	2080	0	0	0	UA
	(0 – 5875)	(300 – 4700)	(0 – 8340)	(0 – 4050)	(0 – 350)	
	*n* = 24	*n* = 6	*n* = 7	*n* = 11	*n* = 3	
EM feature^b^	6/32 (19%)	12/13 (92%)	6/10 (60%)	3/13 (23%)	2/5 (40%)	UA
IIM rash	1/30 (3%)	10/13 (77%)	1/9 (11%)	0/12 (0%)	1/5 (20%)	UA
RP	1/32 (3%)	3/13 (23%)	5/10 (50%)	2/13 (15%)	1/5 (20%)	UA
ILD	1/33 (3%)	3/11 (27%)	2/10 (20%)	1/13 (8%)	0/5 (0%)	UA
Inflammatory joint disease	5/32 (16%)	5/13 (38%)	4/10 (40%)	1/13 (8%)	2/5 (40%)	UA
Myalgia	19/32 (59%)	5/12 (42%)	4/10 (40%)	4/12 (33%)	2/5 (40%)	UA

*^a^At the time of muscle biopsy. ^b^Extramuscular feature: IIM related rash (Heliotrope rash, Gottron’s papules, Gottron’s sign, shawl sign or mechanic’s hands), inflammatory joint disease (polyarthralgia and/or synovitis), ILD or RP. ^c^First recorded assessment post diagnostic biopsy. ^d^At the time of serum collection. CK, creatinine phosphokinase; DM, dermatomyositis; IBM, inclusion body myositis; IIM, idiopathic inflammatory myopathy; ILD, interstitial lung disease; IMNM, immune mediated necrotising myopathy; IU/L, international units per liter; MAA, myositis associated autoantibody; mg, milligrams; MMT8, manual muscle test 8 (0 – 80); MSA, myositis specific autoantibodies; N, number; NSIIM, non-specific idiopathic inflammatory myopathy; PM, polymyositis; PNL, prednisolone; RP, Raynaud’s phenomenon; UA, information unavailable.*

##### Sarcoplasmic HMGB1 levels are elevated in muscle from IIM patients but levels differ according to disease subtype

**FIGURE 1:**
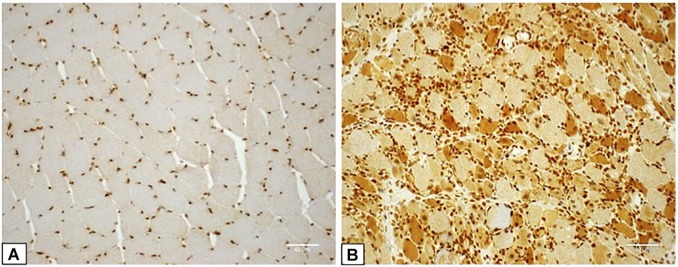
Staining for High Mobility Group Box Protein 1 (HMGB1) in control and IIM muscle. **(A)** Muscle tissue from a control subject demonstrating lightly stippled sarcoplasmic HMGB1 staining and strong positive staining of muscle nuclei. This case was assigned HMGB1 grade zero. **(B)** Muscle tissue from a patient with seronegative MHC I + NM demonstrating positive staining of numerous muscle fibers, muscle cell nuclei and inflammatory cells. This case was assigned HMGB1 grade 3. Magnification: x 200. IIM, idiopathic inflammatory myopathy; IMNM, immune-mediated necrotising myopathy; MHC I, major histocompatibility complex I.

**FIGURE 2:**
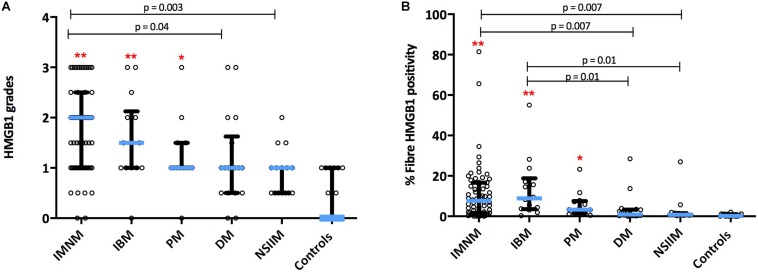
Sarcoplasmic expression of HMGB1 by IIM subtype. Expression levels by **(A)** Grading scales and **(B)** Percentage of positive fibers per high power field. **p < 0.001 versus controls. *p < 0.05 versus controls. DM, dermatomyositis; HMGB1, high mobility group box protein 1; IBM, inclusion body myositis; IIM, idiopathic inflammatory myopathy; IMNM, immune-mediated necrotising myopathy; NSIIM, non-specific idiopathic inflammatory myopathy, PM, polymyositis.

HMGB1 sarcoplasmic immunostaining was low grade and stippled in histologically normal control muscle (Figure 1A) and comparatively strong in IIM patients (Figure 1B). As expected, muscle nuclei and infiltrating immune cells were strongly HMGB1 positive (e.g., Figure 1B). Sarcoplasmic expression was highest in IMNM followed by IBM and PM (Figure 2). Both IBM and IMNM patients exhibited significantly elevated levels of sarcoplasmic staining compared to those with DM and NSIIM.

##### Sarcoplasmic HMGB1 expression correlates with multifactorial processes in IIM

**FIGURE 3:**
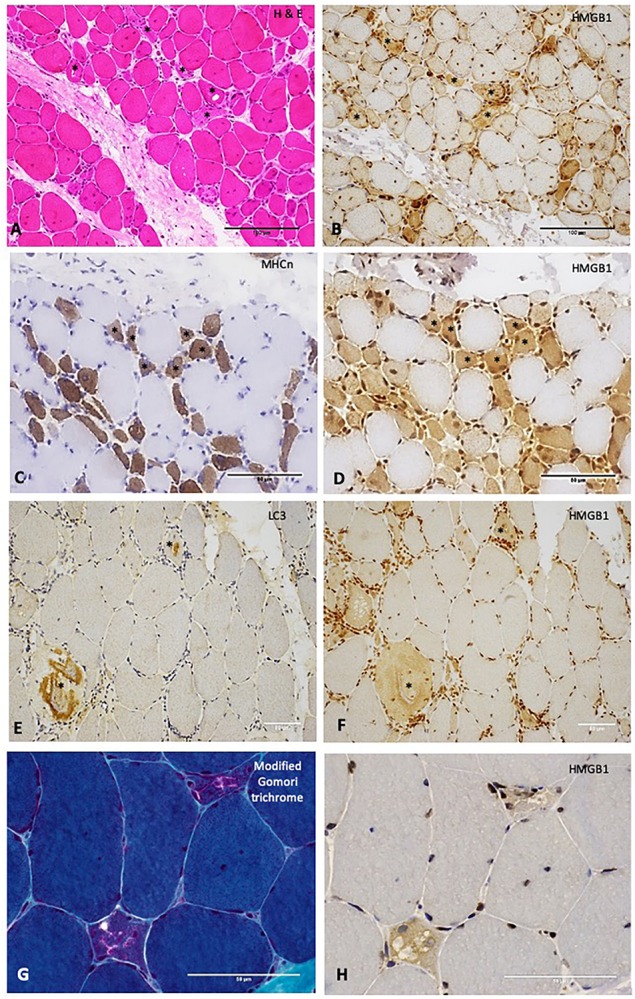
Relationships between High Mobility Group Box Protein 1 (HMGB1) and other histopathological processes in the muscle. **(A)** H&E stain of muscle from a patient with IMNM demonstrating scattered necrotic fibers (examples *). These fibers stain positively for HMGB1 **(B)**. Scale bars 100 microns. **(C)** Neonatal myosin heavy chain (MHCn) staining of a section obtained from a patient with IMNM demonstrating numerous regenerating fibers (examples *). These fibers exhibit strong HMGB1 staining **(D)**. Scale bars 50 microns. **(E)** LC3 staining of muscle from a patient with IBM showing abnormal positivity in two cells (*). These cells were positive for HMGB1 **(F)**. **(G)** Modified Gomori trichrome stain of a patient with IBM demonstrating two cells with abnormal cytoplasmic protein inclusions and vacuolar change. These cells were HMGB1 positive **(H)**. Scale bars 50 microns. Magnification: **(A,B)** x200; **(C–F)** x400; **(G,H)** x 600. HMGB1, high mobility group box protein 1; IMNM, immune-mediated necrotising myopathy; LC3, Microtubule-associated protein 1A/1B-light chain 3.

Sarcoplasmic HMGB1 grades correlated strongly (*r*_s_ 0.62 – 0.77, *p* < 0.01) with the degree of muscle cell necrosis for all IIM subtypes except NSIIM, suggesting that necrosis is an important driver of sarcoplasmic HMGB1 staining even in those subtypes where this is not the dominant histological feature. Both macrophage and CD45+ leucocyte infiltration were strongly associated with sarcoplasmic HMGB1 expression (*r*_s_ 0.63, 0.61, *p* < 0.001), although these correlations were less robust and not significant for IBM patients. In fact, for IBM patients, the degree of MHCn+ (regenerating) fibers (*r*_s_ 0.77, *p* < 0.001) and LC3+ staining (*r*_s_ 0.75, *p* < 0.001) correlated most strongly with HMGB1 expression. On a cellular level, these processes co-localized within individual muscle fibers. Many necrotic cells exhibited strong positive staining for HMGB1 (Figures 3A,B). Regenerating fibers were frequently present in IIM muscle and always stained positively for HMGB1 (Figures 3C,D). In IBM, abnormal HMGB1 staining was visualized in fibers exhibiting features of abnormal cytoplasmic protein inclusions, vacuolar change and autophagic protein accumulation (Figures 3E–H). Isotype control staining was negative (Supplementary Figure 2). Only occasionally was HMGB1 positivity noted in relatively normal appearing mature myofibres. This typically occurred in areas of inflammation and might reflect active secretion of HMGB1 by activated myofibres. Analysis of sarcoplasmic HMGB1 levels by individual autoantibodies was limited by small numbers. Sarcoplasmic HMGB1 levels were high in all anti-SRP+ IMNM patients (Grade ≥ 2, *n* = 5) whereas a range of staining patterns were observed in anti-Ro+ IIM, antisynthetase+ IIM and anti-HMGCR+ IIM.

##### Reduced sarcoplasmic HMGB1 staining in some IIM patients may reflect corticosteroid exposure

We found a modest negative correlation between cumulative corticosteroid dose and sarcoplasmic HMGB1 expression (*r*_s_ −0.30, *p* < 0.01). Patients with inflammatory arthritis had lower muscular HMGB1 staining than those without arthritis (*p* < 0.05). These patients also had higher cumulative prednisolone exposure (325 mg vs. 0 mg, *p* = 0.04), which may explain the comparatively reduced sarcoplasmic HMGB1 expression.

##### Sarcoplasmic HMGB1 correlates with muscle weakness

**FIGURE 4:**
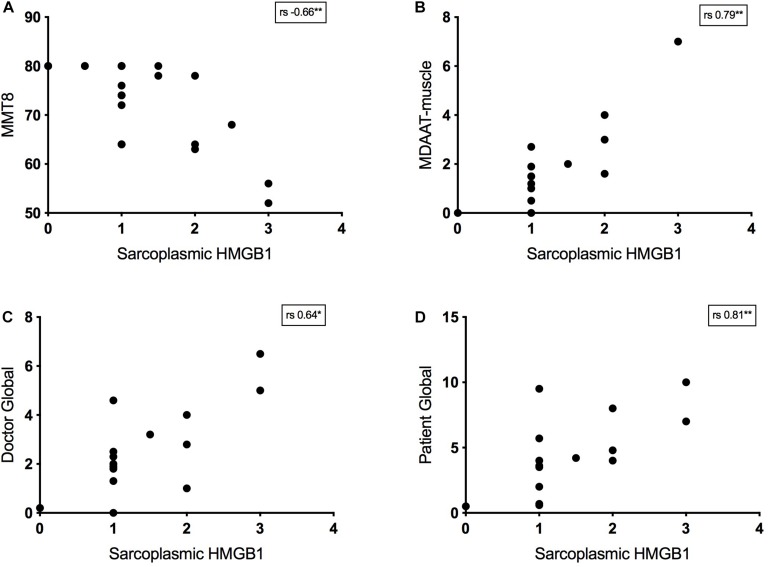
Relationship between bedside disease activity measures and sarcoplasmic HMGB1 grades in IIM patients. Graphs plot HMGB1 grades according to: **(A)** Manual Muscle Testing 8 scores (MMT8, 0–80). **(B)** The muscle activity component of the Myositis Disease Activity Assessment Tool by visual analog scale (MDAAT-muscle VAS, 0 – 10). **(C)** Doctor Global assessment of disease activity by VAS (0 – 10). **(D)** Patient Global assessment of disease activity by VAS (0 – 10).

Twenty-four IIM patients had MMT8 assessments at the time of muscle biopsy and there was a strong negative correlation between strength and sarcoplasmic HMGB1 levels. This was true for IMNM (*r*_s_ −0.57, *p* = 0.03, *n* = 14) and non-IMNM IIM patients (*r*_s_ −0.75, *p* = 0.01, *n* = 19). Sarcoplasmic HMGB1 expression consistently correlated with every bedside clinical disease activity index tested (Figure 4). There was a modest correlation with serum CK level (*r*_s_ 0.31, *p* < 0.01, *n* = 95). There were no correlations between HMGB1 grades and indices of disease-related damage (MDI scores) or patient disability (HAQ scores). Sarcoplasmic HMGB1 expression modestly negatively correlated with symptom duration in IMNM patients (*r*_s_ −0.38, *p* < 0.01, *n* = 50) but trended toward a positive correlation in IBM patients (*r*_s_ 0.62, *p* = 0.07, *n* = 9), likely reflecting that different processes are responsible for HMGB1 expression in these diseases.

##### Serum HMGB1 levels are elevated in patients with PM, DM, and IMNM

**FIGURE 5:**
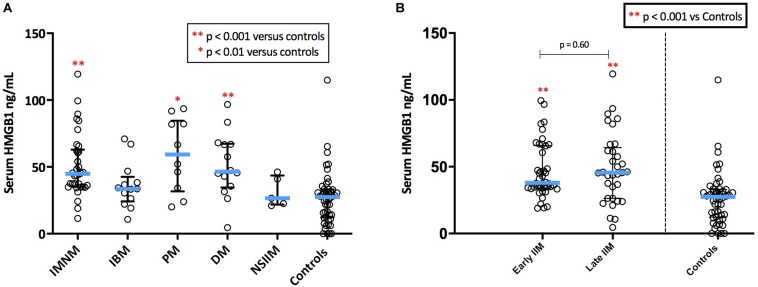
Serum levels of HMGB1 in patients with IIM. **(A)** Serum levels of HMGB1 by IIM subtype. **(B)** Serum levels in IIM patients with early (<6 months since diagnostic biopsy) and late (>6 months since diagnostic biopsy) disease.

Serum HMGB1 was elevated in IIM compared with healthy controls (*p* < 0.001), however, levels varied markedly by IIM subtype (Figure 5A). As such, patterns of serum HMGB1 expression differed from that observed within muscle. For instance, despite exhibiting notable sarcoplasmic staining, circulating levels of HMGB1 in IBM patients did not differ from controls. Conversely, high serum HMGB1 was detected in DM patients despite these patients exhibiting low sarcoplasmic levels. Patients with IMNM had notably high intramuscular and high serum HMGB1 levels.

There were no significant differences in serum HMGB1 levels between IIM patients who had serum collected early in the disease process (within 6 months of diagnosis) and those whose serum collection was more delayed (Figure 5B). There was no correlation between serum HMGB1 levels in IIM patients and symptom duration. Sarcoplasmic and serum levels of HMGB1 did not correlate in the 58 patients with both samples available, possibly owing to the multifactorial nature of intramuscular HMGB1 expression and because these samples were collected at different time-points of the disease trajectory.

##### Extramuscular features are associated with elevated serum HMGB1 levels

High serum HMGB1 levels were observed in IIM patients with RP, ILD and inflammatory joint disease (Table 2). Myalgia was common and was also associated with significantly elevated serum HMGB1. Myalgic patients also had more polyarthralgia (10/33, 30% versus 4/36, 11%, *p* = 0.046) compared with non-myalgic IIM patients, but did not exhibit higher serum CK, myonecrosis or muscle inflammation. Together this suggests that myalgia may be systemically driven or reflect joint inflammation, rather than intramuscular pathology *per se*.

**TABLE 2:** Serum HMGB1 levels according to the presence of certain clinical features.

Clinical feature	Feature present	Feature absent	*p*-value
**Serum HMGB1 (ng/mL) levels by the presence of clinical features**			
ILD	66.96 (36.66 – 83.46) *n* = 7	40.96 (31.46 – 61.29) *n* = 65	0.06
Raynaud’s phenomenon	66.87 (46.81 – 82.81) *n* = 12	37.50 (31.46 – 54.08) *n* = 61	0.001
Inflammatory joint disease	54.08 (35.62 – 67.08) *n* = 17	37.92 (32.25 – 59.10) *n* = 56	0.07
IIM rash	48.50 (35.62 – 68.12) *n* = 13	39.61 (32.51 – 61.64) *n* = 56	0.25
Myalgia	46.11 (36.15 – 69.12) *n* = 34	36.66 (26.26 – 52.00) *n* = 37	0.04
Ro52 antibodies	66.83 (38.25 – 83.46) *n* = 14	40.97 (31.22 – 57.59) *n* = 47	0.02
Antisynthetase antibodies	46.15 (27.04 – 79.37) *n* = 8	45.34 (33.90 – 66.09) *n* = 52	0.88

*IIM, idiopathic inflammatory myopathy; ILD, interstitial lung disease; Ro52, anti-Ro52 antibodies.*

Patients with anti-Ro52+ antibodies had significantly elevated serum HMGB1 levels (Table 2). We did not observe any association between circulating HMGB1 levels and muscle disease activity measures, antisynthetase antibodies or the presence of cancer.

## Discussion

Understanding the molecular events underpinning IIM pathophysiology and how this differs between subtypes is critical in the pursuit of developing targeted therapies, which is increasingly the goal in rheumatological practice. Herein we evaluated expression of HMGB1 in all forms of IIM, including subtypes in which levels of expression of this protein have been hitherto unknown, such as IMNM and NSIIM. Structures demonstrating HMGB1 expression included infiltrating immune cells, necrotic myofibres and those exhibiting regeneration, autophagy and mitochondrial dysfunction. We additionally observed strong HMGB1 staining in all muscle nuclei and low-grade staining in our histologically normal controls. Given small amounts of HMGB1 are present cytosol under normal cellular conditions (Kuehl et al., 1984; Tang et al., 2010), the sarcoplasmic HMGB1 observed in our control tissue is likely physiological. As such, we have shown HMGB1 to be associated with multiple processes in the muscle microenvironment ranging from physiological to pathological, damaging to restorative. The relative balance and temporal evolution of these processes may explain the varying degrees of HMGB1 expression we observed across IIM subtypes. While it seems paradoxical that one protein could be associated with multiple processes, it is in keeping with the complex functional properties of HMGB1. The biological actions of HMGB1 are strikingly diverse and evolve over time owing to its unique biochemistry, its ability to undergo post-translation modifications, complex with other proteins and signal through a multitude of receptors. As others have emphasized (Magna and Pisetsky, 2014), HMGB1 should be conceptualized as an ensemble of proteins rather than a single species and with a fixed structure or function.

Overall, our data imply a deleterious role for HMGB1 in IIM, as sarcoplasmic expression to correlated with clinical disease activity and histological inflammation and necrosis. This is consistent with evidence demonstrating HMGB1 to have pro-inflammatory properties and correlate with inflammatory disease activity. In addition to cytokine and chemokine properties, HMGB1 can activate the classical pathway of complement (Kim et al., 2018), where complement deposition is a key mediator of myonecrosis (Engel and Biesecker, 1982). It is conceivable that HMGB1 released from necrotic myofibres could trigger further local muscle cytolysis and perpetuate further damage in a self-sustaining process. Therapeutic blockade of HMGB1 in inflammatory disease states in is under consideration and been evaluated in experimental models of drug-induced liver injury (Lundback et al., 2016) and inflammatory arthritis (Kokkola et al., 2003), with promising results.

However, data herein and elsewhere links HMGB1 with ostensibly beneficial processes such as myofibre regeneration (De Mori et al., 2007; Tirone et al., 2018) and metabolic functions such as autophagy (Muth et al., 2015; Tang et al., 2010). Accelerated tissue regeneration is observed following administration of recombinant HMGB1 in mouse models of muscle injury (De Mori et al., 2007). This myoregenerative effect is particularly pronounced when HMGB1 is administered in a fully reduced isoform (Tirone et al., 2018). As such, promoting certain HMGB1 pathways therapeutically might accelerate tissue repair in various clinical scenarios. Development of therapeutics that specifically inhibit isoforms of HMGB1 contributing to inflammatory pathology or promote those isoforms involved in reparative processes would clearly be the most desirable strategy. Of note, regenerating myofibres have been implicated in the elaborate pathophysiological mechanisms that underpin IIM (Tournadre and Miossec, 2013), and attempts to enhance myoregeneration with exogenous HMGB1 may not be prudent in these diseases. Further research evaluating the role of specific HMGB1 isoforms in IIM and the role of regenerating myofibres in disease perpetuation is clearly required before therapeutic intervention exploiting HMGB1 pathways can be considered.

We observed elevated serum HMGB1 levels in IMNM, PM and DM patients, however, the source of circulating HMGB1 may differ between subtypes. In IMNM, this likely reflects rapid, passive release of HMGB1 into the extracellular space due to myonecrosis. Indeed, HMGB1 has been used as a marker of necrosis in experimental studies of tumor pathophysiology (Jeon et al., 2013; Kang et al., 2014). However, HMGB1 can also be released from activated immune cells present in inflamed tissues; this may explain the association between HMGB1 levels in serum and the presence of extra-muscular autoimmune manifestations, such as RP, joint disease and ILD. Previous studies have demonstrated elevated HMGB1 in the serum and/or broncho-alveolar fluid of IIM-related ILD and other inflammatory fibrotic lung conditions (Ebina et al., 2011; Shu et al., 2016; Ying et al., 2017; Shimizu et al., 2018). These results support a clinical role for measuring serum HMGB1 levels; this could supplement muscle biopsy in the subtyping of IIM and, potentially, screening for IIM-related ILD (Shu et al., 2016). Our findings suggest that this assessment will have discriminatory utility even in patients with well-established disease who are receiving immunomodulatory therapy, although further studies evaluating the sensitivity and validity of this minimally invasive test are required. Importantly, HMGB1 that is actively secreted by inflammatory cells undergoes critical post-translational modifications (acetylation) of ‘nuclear localization sites’ (NLSs) contained within the protein in order to exit the nucleus (Lotze and Tracey, 2005). Conversely, passive release does not involve NLS modification and thus necrotic cells do not generate hyperacetylated HMGB1 (Yang et al., 2015). An assay that differentiates these HMGB1 isoforms could allow clinicians to quantify the degrees of necrosis versus inflammation in individual patients, and could conceivably aid in IIM subtyping. The oxidation state of HMGB1 also differs according to the mechanism of cellular release but, considering this can rapidly alter in the extracellular milieu (Venereau et al., 2012), assays determining the degree of NLS acetylation may have more discriminatory value. Unfortunately, HMGB1 isoform analysis is challenging, requires high-end mass spectrometry instrumentation and is currently successfully performed by only one research group worldwide on a collaborative basis (Yang et al., 2012). Development of further reliable isoform assays would advance scientific understanding regarding the role of HMGB1 isoforms in disease pathogenesis, information vital for clinical translation of therapeutics targeting HMGB1.

This study has several limitations. It is descriptive in nature and the complex mechanisms underpinning the associations we have observed cannot be determined. We did not perform HMGB1 receptor staining or have access to HMGB1 isoform analysis, which would have provided added insights. Our sample size was small, owing to the rare nature of these disorders. However, this is a large study evaluating HMGB1 expression in IIM and the first to describe elevated levels in the muscle and serum of IMNM patients. These important findings may inform future critical mechanistic studies regarding the role of HMGB1 in autoimmune muscle disorders.

## Data Availability Statement

The datasets generated for this study are available on request to the corresponding author.

## Ethics Statement

This study was approved by the Central Adelaide Local Health Network Ethics Committee, Adelaide, Australia. We confirm that we have read *Muscle and Nerve*’s position on issues involved in ethical publication and affirm that this report is consistent with those guidelines.

## Author Contributions

JD: hypothesis revision, conducting experiments, acquiring and analyzing data, writing manuscript, and acquiring funding. VL: SAMD custodian, hypothesis initiation and revision, acquiring data, manuscript revision, acquiring funding, and providing materials. JH: hypothesis initiation and revision, providing funding, manuscript revision, and providing materials. SP: hypothesis revision, manuscript revision, acquiring data, and providing funding. PE: hypothesis revision, experimental design, and laboratory supervision of JD. PH and SO: hypothesis revision, acquiring data, and providing materials. KC: hypothesis revision, experimental design, conducting experiments, and laboratory supervision of JD. All authors: approval of the final manuscript.

## Conflict of Interest

The authors declare that the research was conducted in the absence of any commercial or financial relationships that could be construed as a potential conflict of interest.

## Abbreviations

ASAb: anti-synthetase antibodies (anti-Jo1, anti-OJ, anti-EJ, anti-PL7, anti-PL12)
CK: creatinine phosphokinase
DM: dermatomyositis
DrG: physician global visual analog scale (0 – 10)
ELISA: enzyme-linked immunosorbent assay
HAQ: Health Assessment Questionnaire (0 – 3)
HMGB1: high mobility group box protein 1
HMGCR: hydroxy-2-methyglutaryl-CoA reductase
HPF: high power field
IBM: inclusion body myositis
IFN: interferon
IIM: idiopathic inflammatory myopathy
ILD: interstitial lung disease
IMACS: International Myositis Assessment and Clinics Studies Group
IMNM: immune mediated necrotising myopathy
IU/L: international units/liter
Jo1: anti-Jo1 antibody
LC3: Microtubule-associated protein 1A/1B-light chain 3
MAA: myositis associated autoantibody
MDAAT-muscle: the muscle activity component of the Myositis Disease Activity Assessment Tool (0 – 10)
MDI: Myositis Damage Index (0 – 10)
mg: milligrams
MHC I: major histocompatibility complex I
MHCn: neonatal myosin heavy chain
MMT8: manual muscle testing 8 (0 – 80)
MSA: myositis specific autoantibodies
N: number
NAM: necrotising autoimmune myopathy
NIMNM: non-immune mediated necrotising myopathy
NSIIM: non-specific idiopathic inflammatory myopathy
PM: polymyositis
PNL: prednisolone
PtG: Patient Global (0 – 10)
Ro: anti-Ro52 antibody
RP: Raynaud’s phenomenon
SAMD: South Australian Myositis Database
SRP: anti-signal recognition peptide antibody
UA: information unavailable
VAS: visual analog scale.

## References

[B1] Allenbach Y. Mammen A. L. Benveniste O. Stenzel W., and Immune-Mediated Necrotizing Myopathies Working G, (2018). 224th ENMC International workshop: clinico-sero-pathological classification of immune-mediated necrotizing myopathies Zandvoort, the Netherlands. *Neuromuscul. Disord.* 28 87–99. 29221629 10.1016/j.nmd.2017.09.016

[B2] Allenbach Y. Benveniste O. (2013). Acquired necrotizing myopathies. *Curr. Opin. Neurol.* 26 554–560. 10.1097/WCO.0b013e328364e9d9 23995277

[B3] Day J. Otto S. Proudman S. Hayball J. D. Limaye V. (2017). Dysregulated innate immune function in the aetiopathogenesis of idiopathic inflammatory myopathies. *Autoimmun. Rev.* 16 87–95. 10.1016/j.autrev.2016.09.019 27666811

[B4] Lotze M. T. Tracey K. J. (2005). High-mobility group box 1 protein (HMGB1): nuclear weapon in the immune arsenal. *Nat. Rev. Immunol.* 5 331–342. 15803152 10.1038/nri1594

[B5] Lu B. Antoine D. J. Kwan K. Lundback P. Wahamaa H. Schierbeck H. (2014). JAK/STAT1 signaling promotes HMGB1 hyperacetylation and nuclear translocation. *Proc. Natl. Acad. Sci. U.S.A.* 111 3068–3073. 10.1073/pnas.1316925111 24469805 PMC3939889

[B6] Gardella S. Andrei C. Ferrera D. Lotti L. V. Torrisi M. R. Bianchi M. E. (2002). The nuclear protein HMGB1 is secreted by monocytes via a non-classical, vesicle-mediated secretory pathway. *EMBO Rep.* 3 995–1001. 12231511 10.1093/embo-reports/kvf198 PMC1307617

[B7] Scaffidi P. Misteli T. Bianchi M. E. (2002). Release of chromatin protein HMGB1 by necrotic cells triggers inflammation. *Nature* 418 191–195. 12110890 10.1038/nature00858

[B8] Harris H. E. Raucci A. (2006). Alarmin(g) news about danger: workshop on innate danger signals and HMGB1. *EMBO Rep.* 7 774–778. 16858429 10.1038/sj.embor.7400759 PMC1525157

[B9] Diener K. R. Al-Dasooqi N. Lousberg E. L. Hayball J. D. (2013). The multifunctional alarmin HMGB1 with roles in the pathophysiology of sepsis and cancer. *Immunol. Cell Biol.* 91 443–450. 10.1038/icb.2013.25 23797067

[B10] Magna M. Pisetsky D. S. (2014). The role of HMGB1 in the pathogenesis of inflammatory and autoimmune diseases. *Mol. Med.* 20 138–146. 10.2119/molmed.2013.00164 24531836 PMC3966993

[B11] Wang H. Qin Y. (2016). Comments on “preliminary study of high mobility group box chromosomal protein 1(HMGB1) in ankylosing spondylitis patients”. *Clin. Exp. Rheumatol.* 34:155. 26517447

[B12] Muth I. E. Zschuntzsch J. Kleinschnitz K. Wrede A. Gerhardt E. Balcarek P. (2015). HMGB1 and RAGE in skeletal muscle inflammation: implications for protein accumulation in inclusion body myositis. *Exp. Neurol.* 271 189–197. 10.1016/j.expneurol.2015.05.023 26048613

[B13] Ulfgren A. K. Grundtman C. Borg K. Alexanderson H. Andersson U. Harris H. E. (2004). Down-regulation of the aberrant expression of the inflammation mediator high mobility group box chromosomal protein 1 in muscle tissue of patients with polymyositis and dermatomyositis treated with corticosteroids. *Arthritis Rheum.* 50 1586–1594. 15146429 10.1002/art.20220

[B14] Grundtman C. Bruton J. Yamada T. Ostberg T. Pisetsky D. S. Harris H. E. (2010). Effects of HMGB1 on in vitro responses of isolated muscle fibers and functional aspects in skeletal muscles of idiopathic inflammatory myopathies. *FASEB J.* 24 570–578. 10.1096/fj.09-144782 19837864

[B15] Cseri K. Vincze J. Cseri J. Fodor J. Csernatony Z. Csernoch L. (2015). HMGB1 expression and muscle regeneration in idiopathic inflammatory myopathies and degenerative joint diseases. *J. Muscle Res. Cell Motil.* 36 255–262. 10.1007/s10974-015-9411-7 25761565

[B16] Shu X. Peng Q. Lu X. Wang G. (2016). HMGB1 may be a biomarker for predicting the outcome in patients with polymyositis/dermatomyositis with interstitial lung disease. *PLoS One.* 11:e0161436. 10.1371/journal.pone.0161436 27537498 PMC4990180

[B17] Zong M. Bruton J. D. Grundtman C. Yang H. Li J. H. Alexanderson H. (2013). TLR4 as receptor for HMGB1 induced muscle dysfunction in myositis. *Ann. Rheum. Dis.* 72 1390–1399. 10.1136/annrheumdis-2012-202207 23148306

[B18] De Mori R. Straino S. Di Carlo A. Mangoni A. Pompilio G. Palumbo R. (2007). Multiple effects of high mobility group box protein 1 in skeletal muscle regeneration. *Arterioscler. Thromb. Vasc. Biol.* 27 2377–2383. 17872450 10.1161/ATVBAHA.107.153429

[B19] Tirone M. Tran N. L. Ceriotti C. Gorzanelli A. Canepari M. Bottinelli R. (2018). High mobility group box 1 orchestrates tissue regeneration via CXCR4. *J. Exp. Med.* 215 303–318. 10.1084/jem.20160217 29203538 PMC5748844

[B20] Tang D. Kang R. Livesey K. M. Cheh C. W. Farkas A. Loughran P. (2010). Endogenous HMGB1 regulates autophagy. *J. Cell Biol.* 190 881–892. 10.1083/jcb.200911078 20819940 PMC2935581

[B21] Cappelletti C. Galbardi B. Kapetis D. Vattemi G. Guglielmi V. Tonin P. (2014). Autophagy, inflammation and innate immunity in inflammatory myopathies. *PLoS One.* 9:e111490. 10.1371/journal.pone.0111490 25365350 PMC4218755

[B22] Limaye V. Luke C. Tucker G. Hill C. Lester S. Blumbergs P. (2013). The incidence and associations of malignancy in a large cohort of patients with biopsy-determined idiopathic inflammatory myositis. *Rheumatol. Int.* 33 965–971. 10.1007/s00296-012-2489-y 22833242

[B23] Rose M. R. Group E. I. W. (2013). 188th ENMC International workshop: inclusion body myositis, 2-4 December 2011, Naarden, the Netherlands. *Neuromuscul. Disord.* 23 1044–1055. 24268584 10.1016/j.nmd.2013.08.007

[B24] Hoogendijk J. E. Amato A. A. Lecky B. R. Choy E. H. Lundberg I. E. Rose M. R. (2004). 119th ENMC international workshop: trial design in adult idiopathic inflammatory myopathies, with the exception of inclusion body myositis, 10-12 October 2003, Naarden, The Netherlands. *Neuromuscul. Disord.* 14 337–345. 15099594 10.1016/j.nmd.2004.02.006

[B25] Cash K. Blumbergs P. (1994). “Neuromuscular tissue,” in *Laboratory Histopathology – A Complete Reference*, eds Woods A. E. Ellis R. C. (New York: Churchill Livingstone), 7.3–25.

[B26] Kuehl L. Salmond B. Tran L. (1984). Concentrations of high-mobility-group proteins in the nucleus and cytoplasm of several rat tissues. *J. Cell Biol.* 99 648–654. 6235236 10.1083/jcb.99.2.648 PMC2113252

[B27] Kim S. Y. Son M. Lee S. E. Park I. H. Kwak M. S. Han M. (2018). High-mobility group box 1-induced complement activation causes sterile inflammation. *Front. Immunol.* 9:705. 10.3389/fimmu.2018.00705 29696019 PMC5904255

[B28] Engel A. G. Biesecker G. (1982). Complement activation in muscle fiber necrosis: demonstration of the membrane attack complex of complement in necrotic fibers. *Ann. Neurol.* 12 289–296. 6753731 10.1002/ana.410120314

[B29] Lundback P. Lea J. D. Sowinska A. Ottosson L. Furst C. M. Steen J. (2016). A novel high mobility group box 1 neutralizing chimeric antibody attenuates drug-induced liver injury and postinjury inflammation in mice. *Hepatology* 64 1699–1710. 10.1002/hep.28736 27474782 PMC5082559

[B30] Kokkola R. Li J. Sundberg E. Aveberger A. C. Palmblad K. Yang H. (2003). Successful treatment of collagen-induced arthritis in mice and rats by targeting extracellular high mobility group box chromosomal protein 1 activity. *Arthritis Rheum.* 48 2052–2058. 12847700 10.1002/art.11161

[B31] Tournadre A. Miossec P. (2013). A critical role for immature muscle precursors in myositis. *Nat. Rev. Rheumatol.* 9 438–442. 10.1038/nrrheum.2013.26 23478496

[B32] Kang R. Chen R. Zhang Q. Hou W. Wu S. Cao L. (2014). HMGB1 in health and disease. *Mol. Aspects Med.* 40 1–116. 10.1016/j.mam.2014.05.001 25010388 PMC4254084

[B33] Jeon H. M. Lee S. Y. Ju M. K. Kim C. H. Park H. G. Kang H. S. (2013). Early growth response 1 regulates glucose deprivation-induced necrosis. *Oncol. Rep.* 29 669–675. 10.3892/or.2012.2134 23152075 PMC3583586

[B34] Ebina M. Taniguchi H. Miyasho T. Yamada S. Shibata N. Ohta H. (2011). Gradual increase of high mobility group protein b1 in the lungs after the onset of acute exacerbation of idiopathic pulmonary fibrosis. *Pulm. Med.* 2011:916486. 10.1155/2011/916486 21637372 PMC3100576

[B35] Ying S. Jiang Z. He X. Yu M. Chen R. Chen J. (2017). Serum HMGB1 as a potential biomarker for patients with asbestos-related diseases. *Dis. Mark.* 2017:5756102. 10.1155/2017/5756102 28348451 PMC5350493

[B36] Shimizu H. Sakamoto S. Isshiki T. Furuya K. Kurosaki A. Homma S. (2018). Association of serum high-mobility group box protein 1 level with outcomes of acute exacerbation of idiopathic pulmonary fibrosis and fibrosing nonspecific interstitial pneumonia. *PLoS One.* 13:e0196558. 10.1371/journal.pone.0196558 29795561 PMC5967827

[B37] Yang H. Wang H. Chavan S. S. Andersson U. (2015). High mobility group box protein 1 (HMGB1): the prototypical endogenous danger molecule. *Mol. Med.* 21(Suppl. 1) S6–S12. 10.2119/molmed.2015.00087 26605648 PMC4661054

[B38] Venereau E. Casalgrandi M. Schiraldi M. Antoine D. J. Cattaneo A. De Marchis F. (2012). Mutually exclusive redox forms of HMGB1 promote cell recruitment or proinflammatory cytokine release. *J. Exp. Med.* 209 1519–1528. 10.1084/jem.20120189 22869893 PMC3428943

[B39] Yang H. Lundback P. Ottosson L. Erlandsson-Harris H. Venereau E. Bianchi M. E. (2012). Redox modification of cysteine residues regulates the cytokine activity of high mobility group box-1 (HMGB1). *Mol. Med.* 18 250–259. 22105604 10.2119/molmed.2011.00389 PMC3324950

