# Supplementary figures and images for: Aberrant Expression of High Mobility Group Box Protein 1 in the Idiopathic Inflammatory Myopathies
Source: Front Cell Dev Biol. 2020 Apr 17;8:226. doi: 10.3389/fcell.2020.00226 (PMC7180187; doi:10.3389/fcell.2020.00226)

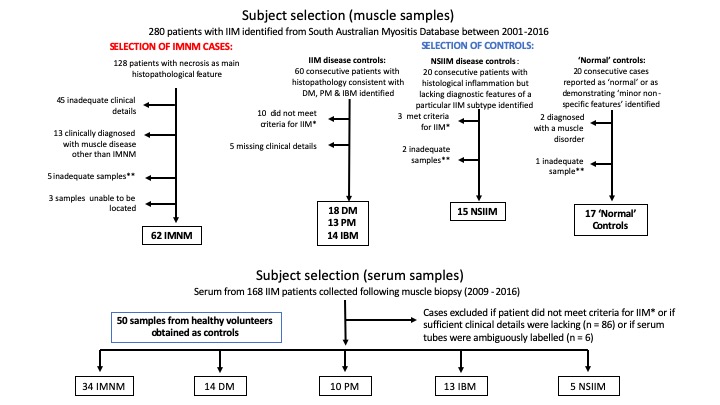

Supplement: Supplementary file 1 [file Image_1.JPEG]

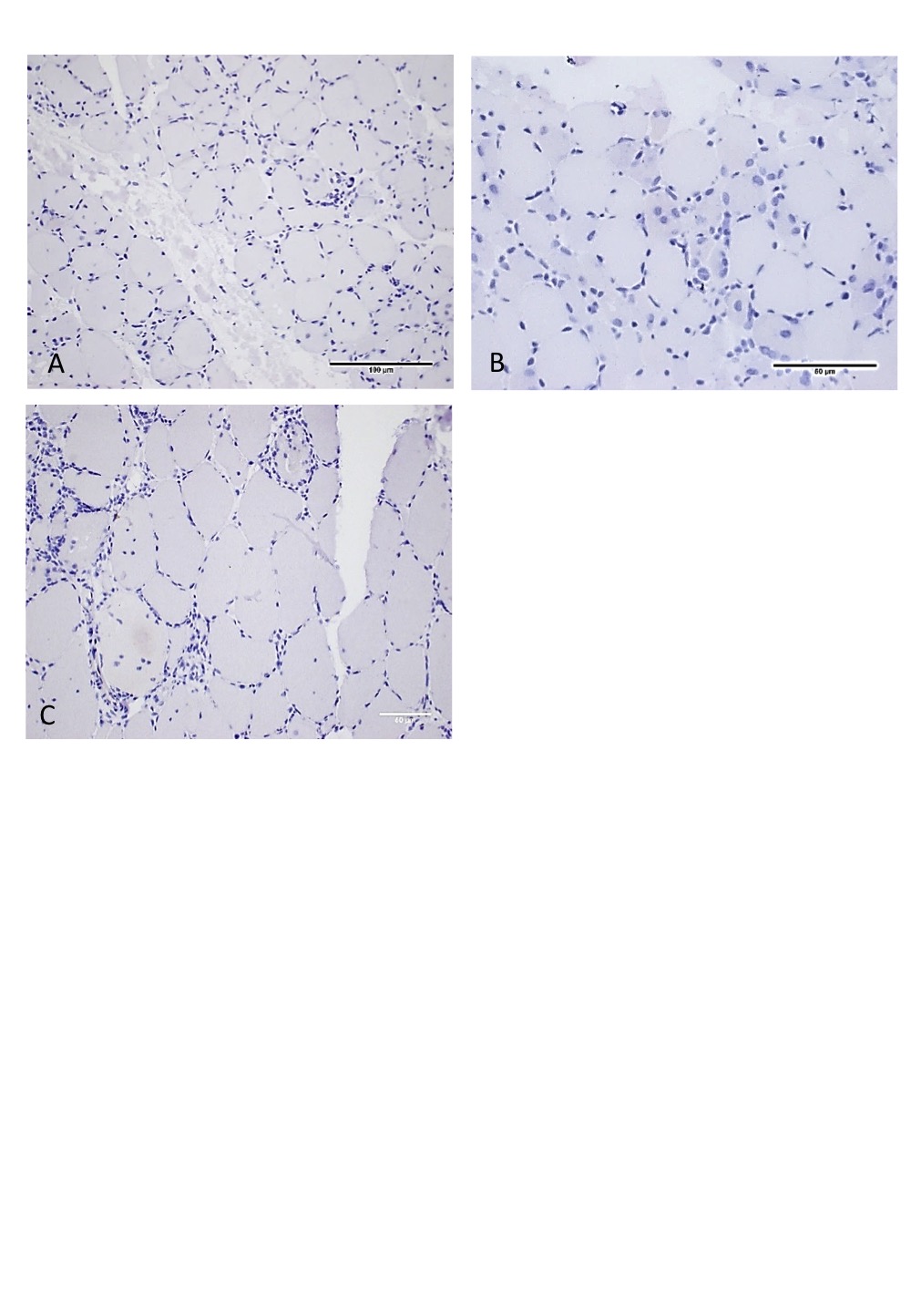

Supplement: Supplementary file 2 [file Image_2.JPEG]
