# Supplementary material for: Aberrant Expression of High Mobility Group Box Protein 1 in the Idiopathic Inflammatory Myopathies
Source: Front Cell Dev Biol. 2020 Apr 17;8:226. doi: 10.3389/fcell.2020.00226 (PMC7180187; doi:10.3389/fcell.2020.00226)
Supplement: Supplementary file 3 [file Table_1.DOCX]

**Supplementary Table 1:** Grading Scales utilised in histopathological evaluation of muscle.

| **Grade** | **Necrosis** | **HMGB1** ^a^ | **MHCn** | **LC3**  **MAC sarcoplasm** | **MHC I ^b^**  **MAC sarcolemmal** |
| --- | --- | --- | --- | --- | --- |
| 0 | No necrotic fibres | No positive muscle fibres | Rare or no positive fibres  [<5%] | Rare or no positive fibres  [<5%] | No sarcolemmal staining |
| 1+ | Small numbers of necrotic fibres  [0-10%] | Small numbers of homogenously positive fibres  [0-10%] | Small numbers of positive fibres  [5-10%] | Small numbers of positive fibres  [5-10%] | Small numbers of fibres with sarcolemmal staining  [0-30%] |
| 2+ | Moderate numbers of necrotic fibres  [11-20%] | Moderate numbers of homogenously positive fibres  [11-30%] | Moderate numbers of positive fibres  [11-30%] | Moderate numbers of positive fibres  [11-30%] | Moderate numbers of fibres with sarcolemmal staining  [30-50%] |
| 3+ | Many necrotic fibres  [20-50%] | Widespread or large focal areas of homogeneously positive fibres  [>30%] | Many positive fibres [>30%] | Many positive fibres [>30%] | Many fibres with sarcolemmal staining [>50%] |
| 4+ | Widespread necrosis  [> 50%] | Not included | Widespread positive fibres  [> 50%] | Not included | Not included |

^a^ Lightly positive sarcoplasmic stippling counted as negative

^b^ Normal appearing fibres graded only

HMGB1, high mobility group box protein 1; LC3, Microtubule-associated protein 1A/1B-light chain 3; MAC, membrane attack complex; MHC I, major histocompatibility complex 1; MHCn, neonatal myosin heavy chain.
