# Supplementary material for: Aberrant Expression of High Mobility Group Box Protein 1 in the Idiopathic Inflammatory Myopathies
Source: Front Cell Dev Biol. 2020 Apr 17;8:226. doi: 10.3389/fcell.2020.00226 (PMC7180187; doi:10.3389/fcell.2020.00226)
Supplement: Supplementary file 4 [file Table_2.DOCX]

**Supplementary Table 2.** Autoantibody profiles of IIM patients according to cohort. Target antigen of autoantibody indicated. Numbers of patients for each autoantibody status indicated, by subtype. Fifty-eight patients (DM = 9, PM = 8, IBM = 7, NSIIM = 5, IMNM = 29) had serum and immunohistochemical analysis performed and hence are represented in both tables.

| **Immunohistochemistry Cohort** | | | | | | |
| --- | --- | --- | --- | --- | --- | --- |
| **IIM Subtype**  **(N tested)** | **Single MSA** | **Single MAA**  **or**  **MAA/MAA combination** | **MSA/MSA combination** | **MSA/MAA combination** | **Euroline negative** | **Euroline testing ND** |
| **DM**  **n = 17** | PL12, n = 1  Mi2, n = 1 | Ro, n = 4  PM/Scl100 and PM/Scl75, n = 1 |  | Ro and Jo1, n = 2  U1RNP and PL12, n = 1 | n = 7 | n = 1 |
| **PM**  **n = 13** | HMGCR, n = 1 | Ro, n = 2  U1RNP, n = 2 |  | Ro and Mi2, n = 1  Ro and Jo, n = 1 | n = 6 |  |
| **IBM**  **n = 12** | PL7, n = 1  PL12, n = 1 | Ro, n = 2 |  |  | n = 8 | n = 2 |
| **IMNM**  **n = 44*** | Mi2, n = 2  HMGCR, n = 8  SRP, n = 3  PL7, n = 2 | Ro, n = 2  PM/Scl75, n = 1 | SRP and PL7, n = 1  HMGCR and PL7, n = 1 | SRP and Ro, n = 1 | n = 23 | n = 18 |
| **NSIIM**  **n = 15** | PL7, n = 1  Jo1, n = 1 | Ro, n = 2  PM/Scl100 and PM/Scl75, n = 1 |  |  | n = 8 | n = 2 |
| **Serum Cohort** | | | | | | |
| **IIM Subtype**  **(N tested)** | **Single MSA** | **Single MAA**  **or**  **MAA/MAA combination** | **MSA/MSA combination** | **MSA/MAA combination** | **Euroline negative** | **Euroline testing ND** |
| **DM**  **n = 13** | Mi2, n = 1  OJ, n = 1 | Ro, n = 4  PM/Scl100 and PM/Scl75, n = 1 |  | Ro and Jo, n = 2 | n = 4 | n = 1 |
| **PM**  **n = 10** |  | Ro, n = 2 |  | Ro and Jo, n = 1  PL7 and Ku, n = 1 | n = 6 |  |
| **IBM**  **n = 10** | PL12, n = 1 | Ro, n = 3 |  |  | n = 6 | n = 3 |
| **IMNM**  **n = 24 #** | HMGCR, n = 4  SRP, n = 2 | Ro, n = 3 | HMGCR and PL7, n = 1 |  | n = 14 | n = 10 |
| **NSIIM**  **n = 5** | PL7, n = 1 | PM/Scl100 and PM/Scl75, n = 1 |  |  | n = 3 |  |

* Only 19 underwent anti-HMGCR ELISA testing; # Only eight underwent anti-HMGCR ELISA testing.

DM, dermatomyositis; HMGCR, anti-3-hydroxy-2-methyglutaryl-CoA reductase antibody; IBM, inclusion body myositis; IIM, idiopathic inflammatory myopathy; IMNM, immune mediated necrotising myopathy; Jo1, anti-Jo1 antibody; Ku, anti-Ku complex antibody; MAA, myositis associated autoantibody; MSA, myositis specific autoantibodies; N, number; ND, not done; NSIIM, non-specific idiopathic inflammatory myopathy; PL7, anti-PL7 antibody; PL12, anti-PL12 antibody; PM, polymyositis; PMSCl75, anti PMScl complex (75kDa antigen) antibody; PMSCl100, anti PMScl complex (100kDa antigen) antibody; Ro, anti-Ro52 antibody; SN, seronegative; SRP, anti signal recognition peptide antibody; U1RNP, anti- U1 ribonucleoprotein antibody.
