# Supplementary material for: Aberrant Expression of High Mobility Group Box Protein 1 in the Idiopathic Inflammatory Myopathies
Source: Front Cell Dev Biol. 2020 Apr 17;8:226. doi: 10.3389/fcell.2020.00226 (PMC7180187; doi:10.3389/fcell.2020.00226)
Supplement: Supplementary file 5 [file Table_3.docx]

**Supplementary Figure 1.** Flow chart of participant selection.

* European Neuromuscular Centre criteria ^23, 24^

** Thawed, damaged or insufficient tissue for sectioning

DM, dermatomyositis; IBM, inclusion body myositis; IIM, idiopathic inflammatory myopathies; IMNM, immune-mediated necrotising myopathy; NSIIM, non-specific idiopathic inflammatory myopathy; PM, polymyositis.

**Supplementary Figure 2.** Isotype control staining of muscle samples revealed no positive staining. **A.** Consecutive sections of muscle depicted in Figure 3A-B. Scale bar 100 microns, magnification x 200. **B**. Consecutive section of muscle depicted in Figure 3C-D. Scale bar 50 microns, magnification x 400. **C**. Consecutive section of muscle depicted in Figure 3E. Scale bar 50 microns, magnification x 400.
